# Influence of Selenium Biofortification of Soybeans on Speciation and Transformation during Seed Germination and Sprouts Quality

**DOI:** 10.3390/foods11091200

**Published:** 2022-04-20

**Authors:** Yatao Huang, Ningyu Lei, Yangyang Xiong, Yanfang Liu, Litao Tong, Fengzhong Wang, Bei Fan, Philippe Maesen, Christophe Blecker

**Affiliations:** 1 Key Laboratory of Agro-Products Quality and Safety Control in Storage and Transport Process, Ministry of Agriculture and Rural Affairs, Institute of Food Science and Technology, Chinese Academy of Agricultural Sciences, Beijing 100193, China; huangyatao@caas.cn (Y.H.); leiny06@163.com (N.L.); 82101215459@caas.cn (Y.X.); liu81340329@163.com (Y.L.); tonglitao@caas.cn (L.T.); fanbei@caas.cn (B.F.); 2 Gembloux Agro-Bio Tech, Department of Food Science and Formulation, TERRA Research Centre, University of Liege, 5030 Gembloux, Belgium; philippe.maesen@uliege.be (P.M.); christophe.blecker@uliege.be (C.B.)

**Keywords:** selenium speciation, soybean, germination, nutrients and bioactive compounds

## Abstract

Selenium (Se) biofortification during seed germination is important not only to meet nutritional demands but also to prevent Se-deficiency-related diseases by producing Se-enriched foods. In this study, we evaluated effects of Se biofortification of soybeans on the Se concentration, speciation, and species transformation as well as nutrients and bioactive compounds in sprouts during germination. Soybean (*Glycine max* L.) seedlings were cultivated in the dark in an incubator with controlled temperature and water conditions and harvested at different time points after soaking in Se solutions (0, 5, 10, 20, 40, and 60 mg/L). Five Se species and main nutrients in the sprouts were determined. The total Se content increased by 87.3 times, and a large portion of inorganic Se was transformed into organic Se during 24 h of germination, with 89.3% of the total Se was bound to soybean protein. Methylselenocysteine (MeSeCys) and selenomethionine (SeMet) were the dominant Se species, MeSeCys decreased during the germination, but SeMet had opposite trend. Se biofortification increased contents of total polyphenol and isoflavonoid compounds and amino acids (both total and essential), especially in low-concentration Se treatment. In conclusion, Se-enriched soybean sprouts have promising potential for Se supplementation and as functional foods.

## 1. Introduction

Selenium (Se) is an essential element with growing interest and plays an important role in the immune system in humans. Chronic Se deficiency may lead to hypothyroidism, subfertility, weakened immunity, and increased possibility of viral infections [1]. Se is beneficial at low levels but toxic at high levels, and the range between deficiency and excess is narrow [2]. According to the recommended dietary allowance, adults should intake Se above 50–60 μg/day [3] but remain below an intake of 400 μg/day. The tolerable upper intake level was recommended by the Food and Nutrition Board of the U.S. Institute of Medicine and the Panel on Dietary Antioxidants and Related Compounds of the National Academies [4]. An estimated one thousand million people have insufficient dietary Se intake [5], especially people living in China, because of the low level of Se in agriculture products [6].

Dietary supplementation has been proven to be a convenient and efficient means of Se supplementation in humans. Se-enriched agro-foods are recognized as the safest way to supplement Se owing to their safety, high quality, and health characteristics [7]. The major techniques used for Se biofortification include genetic fortification, pre-harvest fortification, and post-harvest fortification [8]. Se fortification is achieved using selenate (Se^6+^), selenite (Se^4+^), or selenomethionine (SeMet) and different biofortification methods, including soil supplementation, foliar or fruit spraying, and seed priming [9]. Given its wide global consumption, soybean (*Glycine max* L.) has been often studied as a dietary Se source. Se-enriched soybean may provide Se to the 0.5 to 1 billion people who do not intake enough Se due to dietary inadequacy [10].

Among all biofortification techniques available, fortification during seed germination is a simple, rapid, and efficient method for producing Se-enriched foods [11]. Sprouts can be harvested within 5 to 7 days from the start of germination and are available year-round as highly digestible vegetables such as soybean, potato, radish, broccoli, red clover, alfalfa, kale, and kohlrabi sprouts, which reportedly have a higher Se level than those in unfortified sprouts [12,13,14,15,16]. Soybean sprouts are a nutritious vegetable produced by seed germination, represent a growing market, and have been directly used as a human food for more than 2000 years in oriental cultures [17]. More than 80% of the total Se in soybean is bound to protein, and the Se bioavailability of soybean protein is largely above 90% [11].

Se nutritional activity or toxicity depends not only on its concentration but also on its chemical forms, which originate from various metabolic processes [18]. Further, the mobility and bioavailability of Se also have a close connection with its chemical forms [19]. Foods contain various forms of Se, including selenite and selenate and several seleno amino acids, such as SeMet, methylselenocysteine (MeSeCys), and selenocystine (SeCys_2_). The level of different Se species depends on the crop varieties and the Se treatment technique [4,20]. Because of the low bioavailability and high toxicity of inorganic Se, enriching organic Se in plants through metabolism is important. Therefore, fortification should be considered for increasing the organic speciation of Se. From the perspective of nutrition and safety, the level of organic Se is more important, so the Se speciation and transformation during plant growth is necessary to be understood. As SeMet synthesis is predominantly influenced by the germination time [4], it is important to investigate species changes throughout the germination period; however, as far as we know, few studies have emphasized the changes of Se speciation during soybean germination.

The nutritional values change during Se enrichment of plants should also be emphasized. Se biofortification has been shown to have effects on the concentration of other composition, mainly on amino acids, phenolics, and flavonoids, all of which are important bioactive substances in soybean [21]. A previous study found that application of selenite led to a higher accumulation of selenium in sprouts compared to selenate, which probably results from the faster incorporation of selenite into organic Se compounds in comparison with selenite [22]. The germination of chickpea and soybean sprouts under the treatment of selenite increased the amounts of carotenoids and isoflavones, such as daidzein, genistein, and glycitein, as compared to those in non-selenized sprouts [23,24,25]. The application of Se increased the total phenol contents in coriander and tatsoi microgreens by 21% and 95%, respectively [26]. In extracts of wheat microgreens biofortified with 0.25–0.50 mg/L of Se, bioactive compounds such as phenolics and flavonoids were increased [27]. These previous studies have shown that Se enrichment has effects on secondary plant compounds such as phenolics and flavonoids and that it is possible to maximize multiple bioactive components during soybean germination.

In this study, we analyzed the total Se, Se species, and Se bound to protein during different time of the germination of soybean seeds treated with selenite solutions of various concentrations. As far as we know, it is first time a study has unraveled Se metabolism during soybean sprouts from different perspectives, including species, free, and bound Se, through speciation analysis using high-performance liquid chromatography with inductively coupled plasma mass spectrometry (HPLC-ICP-MS). Nutrients compounds, such as proteins, amino acids, total phenolics, and flavonoids, were analyzed to study how they are affected by Se treatment during germination. We hypothesized that this research would provide novel insights into Se species transformation at various time points during soybean germination, which will help us to obtain specific Se species through germination processing.

## 2. Materials and Methods

### 2.1. Seed Germination and Treatment

Soybean (Zhong Huang 13) seeds were provided by the Institute of Crop Science of CAAS (Beijing, China). Seeds were surface-sterilized with a 0.1% NaClO solution for 5 min, washed with deionized water five times, and soaked in Se solutions of various concentrations (0, 5, 10, 20, 40, and 60 mg/L) at a 1:5 (*w*/*v*) ratio for 6 h. Then, the seeds were transferred into plant tissue culture containers with two layers of gauze. The containers were placed in an incubator with a controlled temperature of 25 °C in the dark. Deionized water was sprayed for 5 s (about 350 mL) every 10 h during germination, and sprouts were harvested every 24 h until 120 h (5 days). The sprouts were freeze-dried, ground with a grinder, and passed through a 40-mesh sieve. Flour samples were stored at −20 °C until analysis.

### 2.2. Chemicals

Total Se and different Se species standard were purchased from the National Standard Material Center (Beijing, China). Deionized water was prepared by a Milli-RO water-purification system (Milli-RO Plus, Molsheim, France). HCl and HNO_3_ were purchased from Beijing Chemical Reagents (Beijing, China). Protease XIV was purchased from Sigma Chemical Co. (Sigma Chemical Co., St. Louis, MO, USA). Other reagents used were purchased from Sinopharm Chemical Reagent Co., Ltd., Beijing, China.

### 2.3. Total Se Determination

Approximately 0.5 g soybean sprout powder was weighed in a 50 mL polypropylene tube, and 6 mL of HNO_3_ and 2 mL of H_2_O_2_ (30%) were added. The mixture was digested at 120 °C until a white fume appeared. Concentrated HCl (5 mL) was added to reduce selenate to selenite. Then, the solution was diluted with deionized water to a volume of 25 mL, and Se was detected by hydride-generation atomic fluorescence spectrometry (HG-AFS 9230; Beijing Titan Instruments, Beijing, China). The limit of quantification and limit of detection for Se-based plant dry weight for the entire procedure were estimated to be 0.48 μg/kg and 0.36 μg/kg, respectively.

### 2.4. Se Species Determination

Samples were prepared according to the previous study [28]: approximately 0.1 g soybean sprout powder was weighed in a 15 mL plastic tube, and 10 mL of Tris-HCl (75 mmol/L, pH 7.5) and 10 mg protease XIV were added. The sample was homogenized by ultrasound at 37 °C for 18 h. Then, it was centrifuged at 10,000 rpm for 30 min. The supernatant was collected and filtered through a 0.22 µm hydrophilic filter, and the filtrate was stored at 4 °C until Se speciation analysis.

Instrumental analysis was conducted according to previous study [29], with some modifications. The different species were determined using an HPLC system (U3000; Thermo Fisher Scientific company, Waltham, MA, USA) equipped with a ZORBAX SB-Aq column (4.6 × 250 mm, particle size, 5 μm). The different species were separated by isocratic elution using a mobile phase (10 mM citric acid, 0.5 mM sodium 1-hexanesulfonate, 2% methanol, pH 5.5) at a flow rate of 0.8 mL/min. The outlet of the HPLC system was coupled to ICP-MS instrument (X Series 2; Thermo Fisher Scientific company, Waltham, MA, USA). The column outlet of HPLC system was connected to Micormist nebulizer using PEEK tubing (0.25 mm i.d. × 10^4^ cm length).

### 2.5. Protein and Amino Acid Determination

Proteins and amino acids were determined as previously reported [30,31]. The total protein concentration of sprout powder was determined using the Kjeldahl method, with a nitrogen-to-protein conversion factor of 6.25. After protein hydrolysis, 17 amino acids were determined using an automatic amino acid analyzer (LA8080; Hitachi, Tokyo, Japan).

### 2.6. Total Phenolic and Total Flavonoid Content Determination

The total phenolic content (TPC) and total flavonoid content (TFC) of the soybean sprout samples were assessed according to a previous report [32]. TPC was determined by the Folin–Ciocalteu assay. All reactions were carried out at room temperature. The results were presented as mg gallic acid equivalent/g dry weight (mg GAE/g DW). All standard dilutions and samples were analyzed in triplicate. TFC was determined by an AlCl_3_ colorimetric assay, and the results are presented by mg catechin equivalent/g dry weight (mg CE/g DW).

### 2.7. Data Analysis

Data were analyzed using one-way and two-way analysis of variance (ANOVA), followed by Duncan’s multiple comparisons test (*p* < 0.05) using the SPSS 19 software (IBM, Armonk, NY, USA). Graphs were generated using Origin 2018 (OriginLab, Northampton, MA, USA).

## 3. Results and Discussion

### 3.1. Total Se Contents in Se-Enriched Soybean Sprouts

The total Se contents in soybean sprouts germinated in the presence of different concentration of Se are shown in Table 1. The total Se content in soybean sprouts was increased by 7.35–87.3 fold upon germination in the presence of 5–60 mg/L sodium selenite solution from 33.03 ± 1.44 μg/kg in the control to 242.5 ± 16.88 μg/kg at 5 mg/L and 2882 ± 86.97 μg/kg at 60 mg/L. These results indicated that germination in the presence of a selenite solution is an efficient method for Se biofortification of soybean sprouts. In China, the National Health Commission estimated that the dietary selenium intake of consumers was 44.60 μg/day [33], which is lower than the RDI (60 μg/day) of selenium, and daily dietary intake of 5.34 to 63.5 g Se-enriched soybean sprouts, for which germination in the presence of 5 to 60 mg/L sodium selenite solution for 120 h could meet the RDI. However, the increments were relatively low compared to previous study, which increased by 2196 fold from 4.6 μg/kg in control to 10,100 μg/kg under 10 mM sodium selenite solution perhaps because they used a different soybean cultivar (Yudou 8) with a low initial Se concentration (4.6 ± 0.5 μg/kg) [11].

The total Se content decreased in the first 24 h of germination and then increased from 24 h to 48 h. This may be because the sodium selenite present on the soybean surface was not absorbed into the soybean tissue in the first 24 h and was washed away during sample preparation. Between 24 h and 48 h, the sodium selenite likely was absorbed into soybean and partly transformed into organic Se. Between 72 h and 120 h, the total Se content decreased again, which was consistent with findings in a previous study in which the highest Se concentrations were noted in grains germinated at 24 °C for 36–48 h [24]. In line herewith, a previous study investigated Se uptake in basil (*Ocimum basilicum* L.) leaves and found that once maximum Se uptake was reached, the Se concentration in the leaves gradually declined, which was attributed to a dilution effect along with plant growth [34]. However, as the soybeans in this study were germinated in the dark, the dry weight decreased from 19.62 ± 0.59 to 17.87 ± 0.40 g, and there was no significant different between Se treatment and control, so the dilution effect cannot explain the above phenomenon. A previous review of the Se accumulation mechanisms during seeding growth showed that young leaves contain relatively high concentrations of Se and exist in the vacuoles of the plant cells, and Se may be transformed into volatilized Se as dimethyl selenide (DMSe) and be effluxed from the vacuole [35]. Therefore, the decrease in total Se could be due to the loss of volatile Se compounds, such as DMeSe and dimethyl diselenide (DMeDSe), which have been found in Se-biofortified plants [36,37]. Thus, when producing Se-enriched soybean sprouts, the time to harvest should be considered to achieve maximum Se uptake.

### 3.2. Se Species in Se-Enriched Soybean Sprouts

**Figure 1:**
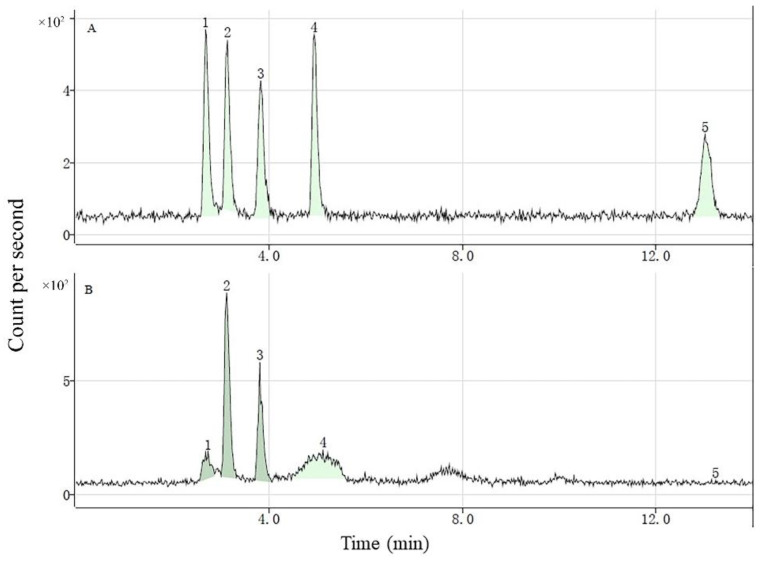
Chromatograms of different Se species of standard solution (5 μg/L) (**A**) and Se-enriched soybean sprouts (**B**). SeCys_2_ (1), MeSeCys (2), SeMet (3), selenite (4), and selenate (5).

HPLC-ICP-MS provided sufficient separation, enabling us to identify the potential Se species in the soybean sprouts (Figure 1). The retention times of standard SeCys_2_, MeSeCys, SeMet, selenite, and selenate were 2.694, 3.146, 3.832, 4.937, and 13.019 min, respectively, and the recovery of the sum of different Se species to total Se were from 70.27% to 120.33%. These five species were determined in the soybean sprouts that were harvested every 24 h, and the majority of organic Se bioaccumulated in the form of MeSeCys and SeMet followed by SeCys_2_, while selenate was not detected in any of the treated sprouts.

**Figure 2:**
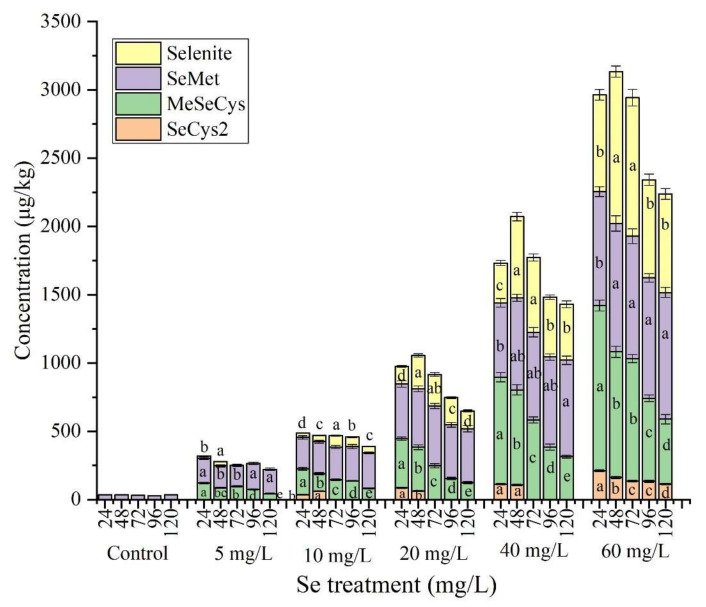
Concentration of Se species under different treatment during the germination. Different letters are used to show significant differences at various treatments (*p* < 0.05) in the same Se species (same color) under different Se concentration treatment, respectively.

The proportion of organic Se (sum of SeCys_2_, MeSeCys, and SeMet) decreased from 86.70% to 79.87% during germination (Figure 2), which was in agreement with findings in a previous study in which the proportion of organic Se rapidly reached a plateau on the second day and then decreased until the ninth day [38]. Selenite was transformed into organic Se during the first 24 h, indicating that germination efficiently transforms inorganic Se into its organic version. With increasing Se concentration of solutions, the organic Se content increased in line with total Se from 221.8 ± 12.04 μg/kg to 2238 ± 78.28 μg/kg, whereas the proportion of organic Se decreased from 100.0% to 67.8%. This may be because the efficiency of selenite assimilation into organic Se decreases when the selenite concentration reaches a certain threshold level, as has been demonstrated in Se-enriched rice [39]. In plants sprayed with a Se solution, the proportion of organic Se was lower than Se enrichment during plant growth regardless of the Se source (selenite or selenate) used [31]; these findings indicate that a longer growth period can promote the formation of organic Se.

Interestingly, the four Se species showed different trends during germination: Selenite increased from 24 h to 48 h and then decreased until 120 h, indicating that part of the selenite was retained on the soybean surface and was washed off during sample preparation. The decrease between 48 h and 120 h can be ascribed to the chemical similarity of Se and S; selenite can be assimilated into SeCys and SeMet and their methylated derivatives after plant uptake [40] as analogues of the sulfur-containing amino acids methionine (Met) and cysteine (Cys) [41]. The SeMet concentration reached 833.8 ± 35.46 μg/kg after 24 h and subsequently increased to 928.4 ± 37.08 μg/kg at 120 h. Similarly, Lazo-Velez et al. found that SeMet synthesis was predominantly influenced by the germination time, and SeMet was synthesized within the first 24 h [42]. An increasing trend in SeMet content has been also found in Se-enriched chlorella [38]. MeSeCys and SeCys_2_ concentrations showed opposite trends with SeMet during germination, as they decreased between 24 h and 120 h. These declines may be due to the biotransformation of MeSeCys and SeCys_2_ into DMeSe and DMeDSe during soybean germination, especially in the late stage. Both DMeSe and DMeDSe are volatile Se compounds that have been also detected in previous studies [36,37]. The Se species changes at different times reveals a useful criterion for the selection of target Se species for Se-enriched soybean sprouts.

### 3.3. Free Se and Bound Se in Se-Enriched Soybean Sprouts

**Figure 3:**
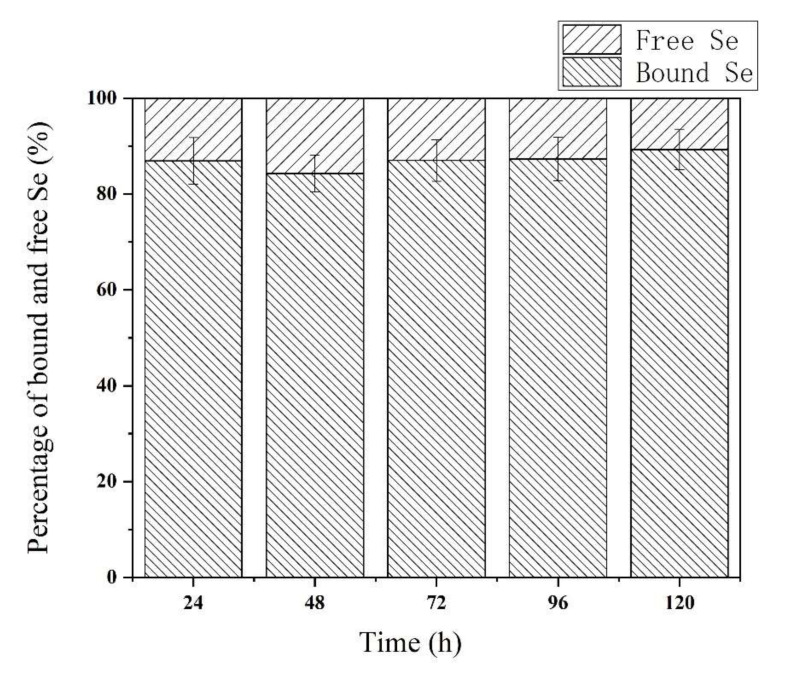
The percentage of bound Se and free Se during soybean germination under 20 mg/L sodium selenite solution.

The free Se species in the soybean sprouts were water-extracted and determined by HPLC-ICP-MS. Se bound to protein, starch, and lipids is difficult to extract with water [39] and was therefore calculated as the difference between the total Se content and the free species content. The results of the free Se species determination are shown in Figure 3. The percentage of free Se to total Se increased from 13.1% at 24 h to 15.7% at 48 h and then decreased to 10.7% at 120 h. The free Se content in this study was lower than that of 35.1% and 22.2% reported for kale and kohlrabi fortified sprouts, respectively [16]. This may be because soybean has more protein than kale and kohlrabi. In contrast, the percentage of bound Se decreased from 86.9% at 24 h to 84.3% at 48 h and then increased to 89.3% at 120 h. Similar results have been reported for soybean planted in Se-enriched soil, where more than 80% Se was incorporated into high-molecular-weight protein [10]. Se is mostly biotransformed into proteins in most Se-enriched plant sources, and in mushroom (*Flammulina velutipes*), the proportion of protein-bound Se increased continually with increasing selenite concentration (0.5–20 μg/g) in the substrate and prolonged harvest time [30]. These findings indicate that Se is not only effectively transformed from inorganic Se to low-molecular-weight seleno metabolites such as seleno amino acids but also incorporated into proteins, as has been previously demonstrated for rice [39].

**Figure 4:**
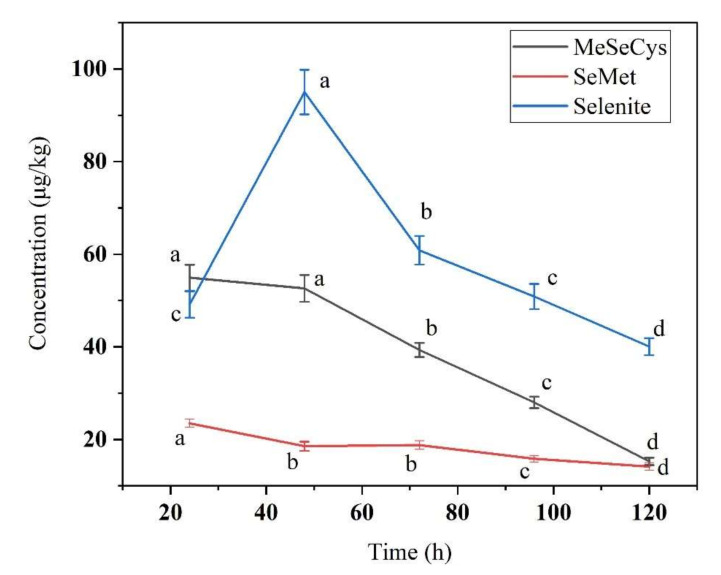
Concentration of three kinds free Se species during soybean germination under 20 mg/L sodium selenite solution. Different letters are used to show significant differences at various treatments (*p* < 0.05) in the same Se species.

**Figure 5:**
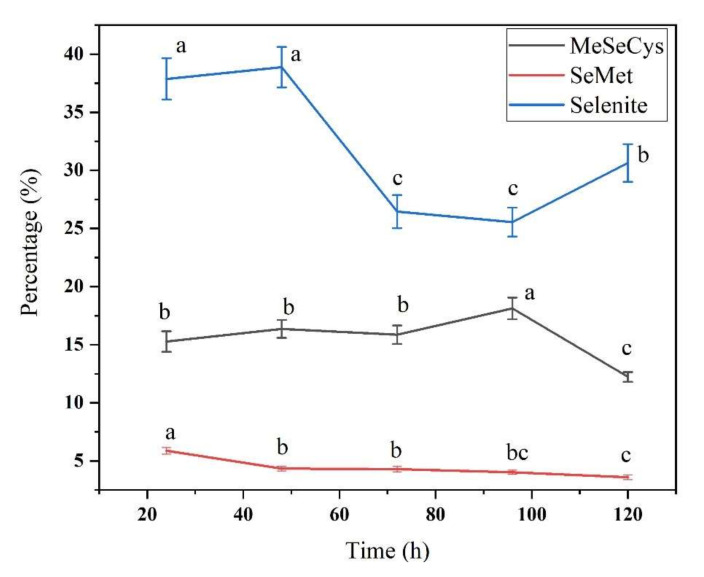
The percentage of three kinds free Se species during soybean germination under 20 mg/L sodium selenite solution. Different letters are used to show significant differences at various treatments (*p* < 0.05) in the same Se species.

We identified the concentration and proportion of three free Se species: selenite, MeSeCys, and SeMet (Figure 4 and Figure 5). The selenite concentration increased from 24 h to 48 h and then decreased until 120 h, and the MeSeCys and SeMet contents decreased from 24 h to 120 h, indicating that inorganic Se is metabolically transformed into different Se species and bound to proteins in the plant tissues. More than 64% of SeMet was bound to protein, and the reason may be that methionine-tRNA cannot distinguish between Met and SeMet, so SeMet incorporated into protein partly in replacement of Met. The replacement of Met with SeMet can disrupt the original protein structure and cause cytotoxicity; however, plants can decrease such damage by regulating the metabolism of different Se species generation [43].

### 3.4. Protein and Amino Acid Compositions in Se-Enriched Soybean Sprouts

The protein content significantly increased during germination (Table 2), which is in line with the findings in a previous study [44], where the crude protein content in soybean seeds was 42.4%, and it increased to approximately 46% during 7 days of germination. There was no significant difference in protein content between the control soybean sprouts and the sprouts treated with various concentrations of Se, possibly because Se concentrations were too low to affect the protein content. Previous studies have shown that Se treatment has different effects on the protein content in plants and that the main factor is the Se treatment dose, as a suitable dose can promote protein synthesis, but at high doses, Se induces various toxicity symptoms and suppresses protein synthesis [30,31,45,46,47].

The amino acid composition in the Se-enriched soybean sprouts is provided in Table 3. The amino acid compositions of the proteins were generally similar among the treatments, which was in agreement with findings in soybeans enriched in Se through foliar spraying [31]. In all treatments, the glutamic acid content was the highest (16.1–17.9%), followed by aspartic acid (15.2–17.8%). This was in agreement with the results of a previous study [48]. The proportion of aspartic acid in the sprouts was higher than that reported for soybeans (6.5–10.0%) because it increased by 106.6% during germination, and it is likely that the increase is due to the hydrolysis of storage protein [48]. Similarly, higher concentrations of aspartic acid have been found after Se treatment of tomato fruit [49].

Under Se treatment, the content of the sulfur-containing amino acid Met decreased from 0.39 ± 0.01 g/100 g to 0.32 ± 0.01 g/100 g, whereas Cys showed no significant difference except at 60 mg/kg Se. This phenomenon has already been observed in Se-enriched soybeans: the Met contents of soybean protein isolate and glycinin in Se-enriched soybeans were considerably lower than those in control soybeans [31], and Met content of proteins was lower in Se-enriched soybeans than in ordinary soybeans [50]. Owing to their physicochemical similarities, Se and sulfur share common metabolic pathways in plants. Specifically, Se uses the sulfur assimilation route, in which it is gradually reduces to selenide and further incorporates into amino acids and then into methylated low-molecular species. As Se and S are chemically similar, the uptake, translocation, and metabolism of Se in plants parallels that of S [9]. The contents of essential amino acids and total amino acids were significantly increased in soybean sprouts treated with Se at concentrations below 40 mg/L and decreased in sprouts treated with Se at concentrations above 60 mg/L, indicating that low-level Se treatment promotes amino acid synthesis, whereas high-level Se treatment has an inhibitory effect. This promotive effect has also been found in Se-enriched golden needle mushroom [30]; in contrast, in Auricularia auricular, Se treatment lowered the total and essential amino acid levels [51]. These contrasting results may be explained by the level of Se tolerance and the detoxification capacity of a plant species [30]. In a comparative proteomics analysis of the effect of Se treatment, 123 differentially expressed proteins were identified, and proteins involved in carbohydrate and amino acid metabolism were the most profoundly affected by Se treatment [52].

### 3.5. TPC and TFC in Se-Enriched Soybean Sprouts

TPC and TFC under Se treatment at 20 mg/L and 60 mg/L are presented in Table 4. Both TPC and TFC increased significantly throughout germination and peaked at 120 h, which is in agreement with previous findings made by our team [53]. The decline at 48 h may be because the hulls, which are rich in polyphenols, fall off at this time point [54]. Both TPC and TFC increased under the two Se treatments when compared to the control treatment. This result was consistent with previous findings of significant increases in phenolics [13] and flavonoids [14] during seed germination under Se treatment and particularly selenite treatment. Notably, with the increase in Se concentration from 20 mg/L to 60 mg/L, there was an increase in TPC but not in TFC. This may be because the flavonoid synthesis is sensitive to Se treatment, and thus, an increasing Se concentration may inhibit flavonoid synthesis. Similar phenomena have been reported in previous studies on wheat and rice [27,55]. Likely, plant phenolic contents increase or decrease depending on the Se treatment concentration and plant species and growth stage. Further, TPC shows variety-specific differences between Golden Delicious and Jonagold, with Jonagold having higher values [56]. Low Se concentrations efficiently elicit the synthesis of phenolic compounds, whereas at higher concentrations, the opposite effect is observed [36]. In germinated chickpeas treated with Se at a low concentration, the phenolics content increased by 19%, whereas at the highest Se concentration, it decreased by 51% compared to that in control sprouts [57]. Germination enhanced the flavonoid content in chickpea by 36%, but under Se treatment, the total phenolic content decreased compared to that in the absence of Se treatment [25]. To unravel the regulation of phenolics biosynthesis under different Se treatments, transcript levels of genes related to phenolics biosynthesis can be investigated. It has been reported that selenite (3 and 6 mg/L) significantly increased the phenolic acid and flavonoid contents in peanut sprouts through phenylpropanoid pathway activation by selenite-induced stress corroborated by phenylalanine ammonia lyase, cinnamate 4-hydroxylase, and 4-coumarate: CoA ligase [58,59].

## 4. Conclusions

The germination of soybean seeds in the presence of selenite resulted in a rapid and substantial accumulation of total Se and organic Se. Compared with root irrigation and foliar spraying, seed soaking before germination is a highly efficient and low-cost technique to produce Se-enriched vegetable sprouts. Total Se increased by 87.3 times, and a large portion of inorganic Se was transformed into organic Se during 24 h of germination. MeSeCys and SeMet were the dominant Se species, but their contents changed during germination, and more than 89% of Se was bound to protein. Protein contents did not significantly differ between Se treatments and the control treatment, whereas the total and essential amino acid contents increased with low-concentration Se treatment and decreased with high-concentration Se treatment. Furthermore, Se biofortification increased TPC and TFC.

Se-enriched soybean sprouts are a promising source for Se supplementation; for example, daily dietary intake of 5.34 g (dry weight) of high Se-enriched soybean sprouts could meet the RDI for Chinese based on current dietary selenium intake. This study provided novel insights into Se species transformation at various time points during soybean germination, which may be useful for the enrichment of soybean sprouts in target Se species. These findings will help us to obtain specific Se species through germination processing.

## Figures and Tables

**Table 1:** Total Se concentration during soybean germination with different concentration Se treatment.

Se Treatment Solution (mg/L)	Total Se Content (μg/kg Dried Weight)					
	0 h	24 h	48 h	72 h	96 h	120 h
Control	33.03 ± 1.44	35.93 ± 1.75	33.44 ± 2.81	33.44 ± 1.31	32.74 ± 2.39	36.12 ± 1.43
5	371.1 ± 15.00 a	302.8 ± 6.63 b	302.8 ± 17.41 b	309.1 ± 21.38 b	284.5 ± 1.18 b	242.5 ± 16.88 c
10	630.6 ± 21.70 a	520.2 ± 26.92 bc	583.0 ± 38.81 ab	613.8 ± 41.18 a	513.5 ± 29.69 bc	446.1 ± 17.73 c
20	1102 ± 30.70 a	1006 ± 43.82 ab	1064 ± 84.71 ab	960.5 ± 24.44 b	806.8 ± 41.42 c	713.7 ± 34.23 c
40	2545 ± 200.4 ab	2217 ± 93.92 bc	2557 ± 81.17 a	2252 ± 100.6 abc	2108 ± 124.3 cd	1880 ± 144.4 d
60	4099 ± 210.6 a	3188 ± 152.2 cd	3426 ± 36.43 bc	3539 ± 125.8 b	2985 ± 135.2 d	2882 ± 86.97 d
Statistical analysis	Se treatment solution (S) ***		Germination hours (G) ***		S × G ***	

The values shown are the mean value for *n* = 3 ± uncertainty (SD). Different letters (a/b/c/d) are used to show significant differences at various treatments (*p* < 0.05) in the same row. *** is used to show significant at *p* < 0.001.

**Table 2:** Total protein concentration during soybean germination with different concentration Se treatment.

Se Treatment Solution (mg/L)	Protein Content (g/100 g Dried Weight)					
	0 h	24 h	48 h	72 h	96 h	120 h
Control	35.32 ± 0.83 c	36.16 ± 0.26 bc	36.69 ± 0.97 abc	37.03 ± 0.80 abc	38.15 ± 0.72 ab	38.68 ± 0.91 a
5	35.24 ± 0.51 d	35.69 ± 0.39 cd	36.34 ± 0.90 cd	36.91 ± 0.61 bc	37.96 ± 0.23 ab	38.40 ± 0.37 a
10	35.63 ± 0.21 b	35.77 ± 0.09 b	36.29 ± 0.58 b	37.00 ± 0.58 ab	37.94 ± 0.84 a	38.01 ± 0.65 a
20	35.78 ± 0.40 c	35.69 ± 0.10 c	36.25 ± 0.36 bc	36.79 ± 0.35 b	38.18 ± 0.11 a	38.46 ± 0.08 a
40	35.52 ± 0.10 c	34.97 ± 0.24 bc	36.21 ± 0.02 b	36.48 ± 0.92 b	38.19 ± 0.12 a	38.29 ± 0.20 a
60	35.38 ± 0.51 c	35.41 ± 0.17 c	35.95 ± 0.07 bc	36.82 ± 0.36 b	37.90 ± 0.59 a	38.14 ± 0.52 a
Statistical analysis	Se treatment solution (S) ns		Germination hours (G) ***		S × G ns	

The values shown are the mean value for *n* = 3 ± uncertainty (SD). Different letters are used to show significant differences at various treatments (*p* < 0.05) in the same row. ns and *** are used to show non-significant and significant at *p* < 0.001.

**Table 3:** Total protein concentration during soybean germination with different concentration Se treatment.

Amino Acids	Se Treatment Solution (mg/L)					
	0	5	10	20	40	60
Aspartic (Asp)	5.82 ± 0.70 a	6.85 ± 0.50 a	6.15 ± 1.12 a	6.23 ± 0.03 a	6.21 ± 0.02 a	4.26 ± 0.00 b
Threonine (Thr)	1.50 ± 0.05 ab	1.47 ± 0.03 ab	1.60 ± 0.07 a	1.57 ± 0.04 ab	1.56 ± 0.11 ab	1.44 ± 0.00 b
Serine (Ser)	2.02 ± 0.07	2.00 ± 0.03	2.15 ± 0.11	2.11 ± 0.03	2.10 ± 0.15	1.95 ± 0.01
Glutamate (Glu)	6.47 ± 0.08 ab	6.19 ± 0.32 b	7.01 ± 0.27 a	6.80 ± 0.05 ab	6.76 ± 0.50 ab	6.62 ± 0.09 ab
Proline (Pro)	1.83 ± 0.05	1.86 ± 0.00	2.03 ± 0.16	1.92 ± 0.01	1.93 ± 0.21	1.86 ± 0.02
Glycine (Gly)	1.54 ± 0.05	1.51 ± 0.03	1.64 ± 0.09	1.61 ± 0.01	1.62 ± 0.13	1.57 ± 0.01
Alanine (Ala)	1.78 ± 0.07	1.77 ± 0.02	1.86 ± 0.13	1.84 ± 0.03	1.86 ± 0.13	1.67 ± 0.02
Cysteine (Cys)	0.39 ± 0.02	0.38 ± 0.02	0.37 ± 0.01	0.38 ± 0.02	0.39 ± 0.01	0.35 ± 0.04
Valine (Val)	1.89 ± 0.07	1.90 ± 0.00	2.00 ± 0.17	1.99 ± 0.03	1.98 ± 0.16	1.90 ± 0.01
Methionine (Met)	0.39 ± 0.01 a	0.34 ± 0.01 b	0.33 ± 0.03 b	0.33 ± 0.01 b	0.31 ± 0.03 b	0.32 ± 0.01 b
Isoleucine (Ile)	1.75 ± 0.03	1.77 ± 0.03	1.88 ± 0.12	1.84 ± 0.05	1.85 ± 0.16	1.80 ± 0.04
Leucine (Leu)	2.87 ± 0.08	2.87 ± 0.05	3.06 ± 0.19	3.01 ± 0.03	3.02 ± 0.21	3.08 ± 0.03
Tyrosine (Tyr)	1.25 ± 0.01	1.27 ± 0.01	1.33 ± 0.12	1.32 ± 0.04	1.35 ± 0.10	1.22 ± 0.01
Phenylalanine (Phe)	1.99 ± 0.04	2.05 ± 0.01	2.15 ± 0.13	2.08 ± 0.05	2.12 ± 0.15	2.06 ± 0.03
Lysine (Lys)	2.52 ± 0.08 a	2.43 ± 0.03 a	2.67 ± 0.15 a	2.51 ± 0.13 a	2.49 ± 0.22 a	1.96 ± 0.01 b
Histidine (His)	1.04 ± 0.05 a	1.08 ± 0.02 a	1.09 ± 0.10 a	1.09 ± 0.04 a	1.09 ± 0.09 a	0.76 ± 0.01 b
Arginine (Arg)	2.69 ± 0.10 a	2.69 ± 0.05 a	2.90 ± 0.18 a	2.80 ± 0.04 a	2.80 ± 0.26 a	2.16 ± 0.01 b
Total amino acids (TAA)	37.74 ± 1.49 ab	38.43 ± 0.05 ab	40.20 ± 3.14 a	39.41 ± 0.64 ab	39.42 ± 2.63 ab	34.98 ± 0.23 b
Total essential amino acids (TEAA)	12.91 ± 0.33 ab	12.84 ± 0.08 ab	13.68 ± 0.85 a	13.32 ± 0.34 ab	13.32 ± 1.03 ab	12.56 ± 0.10 b

The values shown are the mean value for *n* = 2 ± uncertainty (SD). Different letters are used to show significant differences at various treatments (*p* < 0.05) in the same row.

**Table 4:** Total phenolic and total flavonoid content with two different concentrations of Se treatment.

	Se Treatment (mg/L)	0 h	24 h	48 h	72 h	96 h	120 h
TPC (mg GAE/g DW)	Control	0.11 ± 0.014 e	0.11 ± 0.014 e	0.15 ± 0.014 d	0.18 ± 0.007 c	0.31 ± 0.000 b	0.45 ± 0.014 a
	20	0.11 ± 0.014 e	0.12 ± 0.007 e	0.17 ± 0.014 d	0.22 ± 0.007 c	0.42 ± 0.028 b	0.53 ± 0.000 a
	60	0.11 ± 0.014 e	0.12 ± 0.007 e	0.16 ± 0.007 d	0.24 ± 0.007 c	0.46 ± 0.007 b	0.60 ± 0.007 a
Statistical analysis		Se treatment (S) ***		Germination hours (G) ***		S × G ***	
TFC (mg CE/g DW)	Control	7.01 ± 0.04 e	8.95 ± 0.14 d	8.63 ± 0.18 d	9.39 ± 0.13 c	10.28 ± 0.11 b	11.32 ± 0.20 a
	20	7.01 ± 0.04 e	9.29 ± 0.05 d	9.03 ± 0.14 d	9.85 ± 0.20 c	11.75 ± 0.07 b	12.90 ± 0.16 a
	60	7.01 ± 0.04 e	9.21 ± 0.27 d	9.12 ± 0.15 d	9.70 ± 0.07 c	11.00 ± 0.17 b	12.09 ± 0.10 a
Statistical analysis		Se treatment (S) ***		Germination hours (G) ***		S × G ***	

The values shown are the mean value for *n* = 2 ± uncertainty (SD). Different letters are used to show significant differences at various treatments (*p* < 0.05) in the same row. *** is used to show significant at *p* < 0.001.

## Data Availability

The data presented in this study are available upon request from the corresponding author.

## References

[1] Khadra M., Planas D., Brodeur P., Amyot M. (2019). Mercury and Selenium Distribution in Key Tissues and Early Life Stages of Yellow Perch (*Perca flavescens*). Environ. Pollut..

[2] Li Y., Zhu N., Liang X., Zheng L., Zhang C., Li Y.F., Zhang Z., Gao Y., Zhao J. (2020). A Comparative Study on the Accumulation, Translocation and Transformation of Selenite, Selenate, and Senps in a Hydroponic-Plant System. Ecotoxicol. Environ. Saf..

[3] Hatfield D.L., Tsuji P.A., Carlson B.A., Gladyshev V.N. (2014). Selenium and Selenocysteine: Roles in Cancer, Health, and Development. Trends Biochem. Sci..

[4] Pyrzynska K., Sentkowska A. (2021). Selenium in Plant Foods: Speciation Analysis, Bioavailability, and Factors Affecting Composition. Crit. Rev. Food Sci. Nutr..

[5] Adadi P., Barakova N.V., Muravyov K.Y., Krivoshapkina E.F. (2019). Designing Selenium Functional Foods and Beverages: A Review. Food Res. Int..

[6] Kipp A.P., Strohm D., Brigelius-Flohe R., Schomburg L., Bechthold A., Leschik-Bonnet E., Heseker H., Society German Nutrition (2015). Revised Reference Values for Selenium Intake. J. Trace Elem. Med. Biol..

[7] Bermingham E.N., Hesketh J.E., Sinclair B.R., Koolaard J.P., Roy N.C. (2014). Selenium-Enriched Foods Are More Effective at Increasing Glutathione Peroxidase (Gpx) Activity Compared with Selenomethionine: A Meta-Analysis. Nutrients.

[8] Saha S., Roy A. (2020). Whole Grain Rice Fortification as a Solution to Micronutrient Deficiency: Technologies and Need for More Viable Alternatives. Food Chem..

[9] Puccinelli M., Malorgio F., Pezzarossa B. (2017). Selenium Enrichment of Horticultural Crops. Molecules.

[10] Chan Q., Afton S.E., Caruso J.A. (2010). Selenium Speciation Profiles in Selenite-Enriched Soybean (*Glycine max*) by Hplc-Icpms and Esi-Itms. Metallomics.

[11] Deng B., Tian S., Li S., Guo M., Liu H., Li Y., Wang Q., Zhao X. (2020). A Simple, Rapid and Efficient Method for Essential Element Supplementation Based on Seed Germination. Food Chem..

[12] Trolove S.N., Tan Y., Morrison S.C., Feng L., Eason J. (2018). Development of a Method for Producing Selenium-Enriched Radish Sprouts. LWT.

[13] Chiriac E.R., Chiţescu C.L., Sandru C., Geană E.I., Lupoae M., Dobre M., Borda D., Gird C.E., Boscencu R. (2020). Comparative Study of the Bioactive Properties and Elemental Composition of Red Clover (*Trifolium pratense*) and Alfalfa (*Medicago sativa*) Sprouts during Germination. Appl. Sci..

[14] Tian M., Xu X., Liu Y., Xie L., Pan S. (2016). Effect of Se Treatment on Glucosinolate Metabolism and Health-Promoting Compounds in the Broccoli Sprouts of Three Cultivars. Food Chem..

[15] Nurminsky V.N., Perfileva A.I., Kapustina I.S., Graskova I.A., Sukhov B.G., Trofimov B.A. (2020). Growth-Stimulating Activity of Natural Polymer-Based Nanocomposites of Selenium during the Germination of Cultivated Plant Seeds. Dokl. Biochem. Biophys..

[16] Zagrodzki P., Pasko P., Galanty A., Tyszka-Czochara M., Wietecha-Posluszny R., Rubio P.S., Barton H., Prochownik E., Muszynska B., Sulkowska-Ziaja K. (2020). Does Selenium Fortification of Kale and Kohlrabi Sprouts Change Significantly Their Biochemical and Cytotoxic Properties?. J. Trace Elem. Med. Biol..

[17] Guo Y., Chen H., Song Y., Gu Z. (2011). Effects of Soaking and Aeration Treatment on Γ-Aminobutyric Acid Accumulation in Germinated Soybean (*Glycine max* L.). Eur. Food Res. Technol..

[18] Umysova D., Vitova M., Douskova I., Bisova K., Hlavova M., Cizkova M., Machat J., Doucha J., Zachleder V. (2009). Bioaccumulation and Toxicity of Selenium Compounds in the Green Alga Scenedesmus Quadricauda. BMC Plant Biol..

[19] Pyrzynska K. (2020). Nanomaterials in Speciation Analysis of Metals and Metalloids. Talanta.

[20] Hu T., Li H., Zhao G., Guo Y. (2021). Selenium Enriched Hypsizygus Marmoreus, a Potential Food Supplement with Improved Se Bioavailability. LWT.

[21] Cui M., Wu D., Bao K., Wen Z., Hao Y., Luo L. (2019). Dynamic Changes of Phenolic Compounds during Artificial Aging of Soybean Seeds Identified by High-Performance Liquid Chromatography Coupled with Transcript Analysis. Anal. Bioanal. Chem..

[22] Woch W., Hawrylak-Nowak B. (2019). Selected Antioxidant Properties of Alfalfa, Radish, and White Mustard Sprouts Biofortified with Selenium. Acta Agrobot..

[23] Guardado-Felix D., Serna-Saldivar S.O., Cuevas-Rodriguez E.O., Jacobo-Velazquez D.A., Gutierrez-Uribe J.A. (2017). Effect of Sodium Selenite on Isoflavonoid Contents and Antioxidant Capacity of Chickpea (*Cicer arietinum* L.) Sprouts. Food Chem..

[24] Lazo-Vélez M.A., Guardado-Félix D., Avilés-González J., Romo-López I., Serna-Saldívar S.O. (2018). Effect of Germination with Sodium Selenite on the Isoflavones and Cellular Antioxidant Activity of Soybean (*Glycine max*). LWT.

[25] Guardado-Felix D., Serna-Saldivar S.O., Gutierrez-Uribe J.A., Chuck-Hernandez C. (2019). Selenium in Germinated Chickpea (*Cicer arietinum* L.) Increases the Stability of Its Oil Fraction. Plants.

[26] Pannico A., El-Nakhel C., Graziani G., Kyriacou M.C., Giordano M., Soteriou G.A., Zarrelli A., Ritieni A., Pascale S., Rouphael Y. (2020). Selenium Biofortification Impacts the Nutritive Value, Polyphenolic Content, and Bioactive Constitution of Variable Microgreens Genotypes. Antioxidants.

[27] Islam M.Z., Park B.J., Kang H.M., Lee Y.T. (2020). Influence of Selenium Biofortification on the Bioactive Compounds and Antioxidant Activity of Wheat Microgreen Extract. Food Chem..

[28] Luo L., Zhang J., Zhang K., Wen Q., Ming K., Xiong H., Ning F. (2021). Peanut Selenium Distribution, Concentration, Speciation, and Effects on Proteins after Exogenous Selenium Biofortification. Food Chem..

[29] Zhang K., Guo X., Zhao Q., Han Y., Zhan T., Li Y., Tang C., Zhang J. (2020). Development and Application of a HPLC-ICP-MS Method to Determine Selenium Speciation in Muscle of Pigs Treated with Different Selenium Supplements. Food Chem..

[30] Dong Z., Xiao Y., Wu H. (2021). Selenium Accumulation, Speciation, and Its Effect on Nutritive Value of Flammulina Velutipes (Golden Needle Mushroom). Food Chem..

[31] Deng X., Liao J., Zhao Z., Qin Y., Liu X. (2021). Distribution and Speciation of Selenium in Soybean Proteins and Its Effect on Protein Structure and Functionality. Food Chem..

[32] Wu T., McCallum J.L., Wang S., Liu R., Zhu H., Tsao R. (2013). Evaluation of Antioxidant Activities and Chemical Characterisation of Staghorn Sumac Fruit (*Rhus hirta* L.). Food Chem..

[33] Wang J., Yang L., Li H., Li Y., Wei B. (2018). Dietary Selenium Intake Based on the Chinese Food Pagoda: The Influence of Dietary Patterns on Selenium Intake. Nutr. J..

[34] Puccinelli M., Malorgio F., Rosellini I., Pezzarossa B. (2017). Uptake and Partitioning of Selenium in Basil (*Ocimum basilicum* L.) Plants Grown in Hydroponics. Sci. Hortic..

[35] Hossain A., Skalicky M., Brestic M., Maitra S., Sarkar S., Ahmad Z., Vemuri H., Garai S., Mondal M., Bhatt R. (2021). Selenium Biofortification: Roles, Mechanisms, Responses and Prospects. Molecules.

[36] Gupta M., Gupta S. (2016). An Overview of Selenium Uptake, Metabolism, and Toxicity in Plants. Front. Plant Sci..

[37] Moreno-Martin G., Sanz-Landaluze J., Leon-Gonzalez M.E., Madrid Y. (2019). In-Vivo Solid Phase Microextraction for Quantitative Analysis of Volatile Organoselenium Compounds in Plants. Anal. Chim. Acta.

[38] Mylenko M., Vu D.L., Kuta J., Ranglova K., Kubac D., Lakatos G., Grivalsky T., Caporgno M.P., da Camara Manoel J.A., Kopecky J. (2020). Selenium Incorporation to Amino Acids in Chlorella Cultures Grown in Phototrophic and Heterotrophic Regimes. J. Agric. Food Chem..

[39] Hu Z., Cheng Y., Suzuki N., Guo X., Xiong H., Ogra Y. (2018). Speciation of Selenium in Brown Rice Fertilized with Selenite and Effects of Selenium Fertilization on Rice Proteins. Int. J. Mol. Sci..

[40] Schiavon M., Ertani A., Parrasia S., Vecchia F.D. (2017). Selenium Accumulation and Metabolism in Algae. Aquat. Toxicol..

[41] Kieliszek M., Bierla K., Jimenez-Lamana J., Kot A.M., Alcantara-Duran J., Piwowarek K., Blazejak S., Szpunar J. (2020). Metabolic Response of the Yeast Candida Utilis during Enrichment in Selenium. Int. J. Mol. Sci..

[42] Lazo-Velez M.A., Aviles-Gonzalez J., Serna-Saldivar S.O., Temblador-Perez M.C. (2016). Optimization of Wheat Sprouting for Production of Selenium Enriched Kernels Using Response Surface Methodology and Desirability Function. LWT.

[43] Kieliszek M., Blazejak S., Bzducha-Wrobel A., Kot A.M. (2019). Effect of Selenium on Growth and Antioxidative System of Yeast Cells. Mol. Biol. Rep..

[44] Shi H., Nam P.K., Ma Y. (2010). Comprehensive Profiling of Isoflavones, Phytosterols, Tocopherols, Minerals, Crude Protein, Lipid, and Sugar during Soybean (*Glycine max*) Germination. J. Agric. Food Chem..

[45] Luo H., Du B., He L., Zheng A., Pan S., Tang X. (2019). Foliar Application of Sodium Selenate Induces Regulation in Yield Formation, Grain Quality Characters and 2-Acetyl-1-Pyrroline Biosynthesis in Fragrant Rice. BMC Plant Biol..

[46] Dong Z., Lin Y., Wu H., Zhang M. (2021). Selenium Accumulation in Protein Fractions of Tenebrio Molitor Larvae and the Antioxidant and Immunoregulatory Activity of Protein Hydrolysates. Food Chem..

[47] Wrobel K., Guerrero Esperanza M., Yanez Barrientos E., Corrales Escobosa A.R., Wrobel K. (2020). Different Approaches in Metabolomic Analysis of Plants Exposed to Selenium: A Comprehensive Review. Acta Physiol. Plant..

[48] Sun W.X., Zhang R.J., Fan J., He Y., Mao X.H. (2018). Comprehensive Transformative Profiling of Nutritional and Functional Constituents during Germination of Soybean Sprouts. J. Food Meas. Charact..

[49] Zhu Z., Zhang Y., Liu J., Chen Y., Zhang X. (2018). Exploring the Effects of Selenium Treatment on the Nutritional Quality of Tomato Fruit. Food Chem..

[50] Zhao X., Zhao Q., Chen H., Xiong H. (2019). Distribution and Effects of Natural Selenium in Soybean Proteins and Its Protective Role in Soybean Beta-Conglycinin (7s Globulins) under Aaph-Induced Oxidative Stress. Food. Chem..

[51] Hu T., Li L., Hui G., Zhang J., Li H., Wu W., Wei X., Guo Y. (2019). Selenium Biofortification and Its Effect on Multi-Element Change in Auricularia Auricular. Food Chem..

[52] Liang K., Liang S., Zhu H. (2020). Comparative Proteomics Analysis of the Effect of Selenium Treatment on the Quality of Foxtail Millet. LWT.

[53] Wang F., Wang H., Wang D., Fang F., Lai J., Wu T., Tsao R. (2015). Isoflavone, Γ-Aminobutyric Acid Contents and Antioxidant Activities Are Significantly Increased during Germination of Three Chinese Soybean Cultivars. J. Funct. Foods.

[54] Cabezudo I., Meini M.R., Di Ponte C.C., Melnichuk N., Boschetti C.E., Romanini D. (2021). Soybean (*Glycine max*) Hull Valorization through the Extraction of Polyphenols by Green Alternative Methods. Food Chem..

[55] D’Amato R., Fontanella M.C., Falcinelli B., Beone G.M., Bravi E., Marconi O., Benincasa P., Businelli D. (2018). Selenium Biofortification in Rice (*Oryza sativa* L.) Sprouting: Effects on Se Yield and Nutritional Traits with Focus on Phenolic Acid Profile. J. Agric. Food Chem..

[56] Groth S., Budke C., Neugart S., Ackermann S., Kappenstein F.S., Daum D., Rohn S. (2020). Influence of a Selenium Biofortification on Antioxidant Properties and Phenolic Compounds of Apples (*Malus domestica*). Antioxidants.

[57] Serrano-Sandoval S.N., Guardado-Félix D., Gutiérrez-Uribe J.A. (2022). Deglycosylation of Isoflavones in Selenized Germinated Chickpea Flours Due to Convection Drying. LWT.

[58] Sharma A., Shahzad B., Rehman A., Bhardwaj R., Landi M., Zheng B. (2019). Response of Phenylpropanoid Pathway and the Role of Polyphenols in Plants under Abiotic Stress. Molecules.

[59] Ma M., Wang P., Yang R., Zhou T., Gu Z. (2019). Uv-B Mediates Isoflavone Accumulation and Oxidative-Antioxidant System Responses in Germinating Soybean. Food Chem..

